# Metabolic clearance of select opioids and opioid antagonists using hepatic spheroids and recombinant cytochrome P450 enzymes

**DOI:** 10.1002/prp2.1000

**Published:** 2022-08-31

**Authors:** Wing Y. Tuet, Samuel A. Pierce, Matthieu Conroy, Justin N. Vignola, Justin Tressler, Robert C. diTargiani, Bryan J. McCranor, Benjamin Wong

**Affiliations:** ^1^ Pharmaceutical Sciences Department US Army Medical Research Institute of Chemical Defense Aberdeen Proving Ground Maryland USA

## Abstract

The opioid crisis is a pressing public health issue, exacerbated by the emergence of more potent synthetic opioids, particularly fentanyl and its analogs. While competitive antagonists exist, their efficacy against synthetic opioids is largely unknown. Furthermore, due to the short durations of action of current antagonists, renarcotization remains a concern. In this study, metabolic activity was characterized for fentanyl‐class opioids and common opioid antagonists using multiple in vitro systems, namely, cytochrome P450 (CYP) enzymes and hepatic spheroids, after which an in vitro*‐*in vivo correlation was applied to convert in vitro metabolic activity to predictive in vivo intrinsic clearance. For all substrates, intrinsic hepatic metabolism was higher than the composite of CYP activities, due to fundamental differences between whole cells and single enzymatic reactions. Of the CYP isozymes investigated, 3A4 yielded the highest absolute and relative metabolism across all substrates, with largely negligible contributions from 2D6 and 2C19. Comparative analysis highlighted elevated lipophilicity and diminished CYP3A4 activity as potential considerations for the development of more efficacious opioid antagonists. Finally, antagonists with a high degree of molecular similarity exhibited comparable clearance, providing a basis for structure‐metabolism relationships. Together, these results provide multiple screening criteria for early stage drug discovery involving opioid countermeasures.

## Graphical abstract

This study represents the first characterization of metabolic clearance for multiple common opioid antagonists and fentanyl‐class opioids using the same in vitro systems. The direct comparability enables a thorough understanding of the relative stability of opioids and opioid antagonists for the development of more efficacious countermeasures, and measured metabolic clearances can be incorporated into pharmacokinetic models.

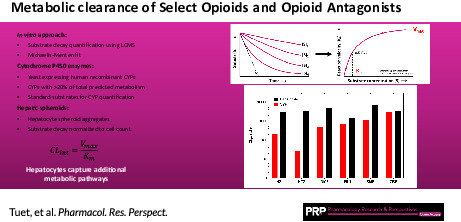

**Abbreviations**
CE: collision energy
CL_H_
: hepatic clearance
CL_int_
: intrinsic clearance
CRF: carfentanil
CXP: collision exit potential
DM: dextromethorphan
FEN: fentanyl
*f*_u,p_
: fraction of unbound drug in the plasma
IVIVC: in vitro‐in vivo correlation
MRM: multiple reaction monitoring
NMF: nalmefene
NTX: naltrexone
NX: naloxone
RMF: remifentanil
SM: S‐mephenytoin
SPE: solid phase extraction
T_c_
: Tanimoto coefficient
TES: testosterone
*t*_*R*_
: retention time

## 1 INTRODUCTION

Opioid abuse is a global public health crisis with devastating outcomes. Incidences of opioid‐induced fatalities have been regularly reported and have risen significantly over the past two decades.^1^, ^2^, ^3^ This development is due, in part, to the emergence and growing prevalence of synthetic opioids with faster onsets of action and higher binding affinities.^4^, ^5^ Of particular interest are fentanyl (FEN) and its analogs, which are estimated to be hundreds to thousands of times more potent than morphine and together accounted for approximately 20% of all opioid‐related deaths in the U.S. during 2016–2017.^6^, ^7^ Use of FEN‐class opioids as a potential lethal agent was also underscored by the 2002 Moscow theater incident, where an aerosol mixture of carfentanil (CRF) and remifentanil (RMF) was introduced into the ventilation system in an attempt to manage a hostage situation, resulting instead in 125 deaths.^8^, ^9^ These concerning trends highlight the severity of the opioid crisis and emphasize the need to better understand the pharmacokinetics of opioids and potential therapeutics.

Currently, competitive antagonists that are effective against morphine and FEN exist. The most extensively studied of these include naloxone (NX), naltrexone (NTX), and nalmefene (NMF), all of which have been shown to reverse opioid‐induced respiratory depression in humans and non‐human species.^10^, ^11^, ^12^, ^13^, ^14^ However, the therapeutic potential of current antagonists against more potent synthetic opioids remains largely unknown.^15^, ^16^, ^17^, ^18^ Renarcotization, wherein opioid‐ induced effects reappear following countermeasure administration and apparent recovery, represents an ongoing concern, particularly for more potent opioids, as current approved antagonists are often characterized by relatively short durations of action.^19^, ^20^ Notably, episodes of renarcotization have been reported for NTX, even though it is inherently longer‐acting and more potent than NX.^21^ This possibility of renarcotization necessitates a more comprehensive understanding of the metabolic stability of opioids and opioid antagonists, especially in relation to each other.

Metabolism of drugs can occur via a variety of biotransformation processes (e.g., oxidation, hydrolysis, conjugation), although the ultimate goal is always to increase hydrophilicity and facilitate excretion from the body.^22^ In general, drug metabolism can be further differentiated into two phases, phase I modification and phase II conjugation, with both phases primarily localized in the liver.^23^ The majority of opioids are metabolized extensively via phase I oxidation by cytochrome P450 enzymes (CYPs), a superfamily of enzymes involved in approximately 75% of all marketed drug metabolism.^24^, ^25^ Current opioid antagonists, on the other hand, are thought to undergo phase II glucuronidation more readily, likely due to their structural similarity to morphine.^25^, ^26^ These differences are important distinctions for metabolic investigations as commonly used in vitro models can contain diverse amalgamations of metabolic pathways, and not all models include relevant transport phenomena that influence drug uptake.^27^, ^28^, ^29^ In theory, intrinsic clearances should be equivalent after physiological scaling; however, several fold differences have been observed between intrinsic clearances as determined using different in vitro systems or different donors within a single in vitro system.^30^, ^31^, ^32^ As such, while metabolic clearance has been studied for specific opioids and opioid antagonists, multiple in vitro systems were employed, which hinders the ability to directly compare between studies.^32^, ^33^, ^34^

In the present study, metabolic clearance was characterized for FEN‐class opioids (FEN, RMF, CRF) and common opioid antagonists (NX, NTX, NMF). Individual recombinant CYPs and hepatic spheroids were investigated to determine contributions of individual metabolic routes and total metabolism, respectively. Substrate consumption was quantified using liquid chromatography with tandem mass spectrometry (LC–MS/MS), and Michaelis–Menten parameters were derived from initial reaction velocities. Finally, an in vitro‐in vivo correlation (IVIVC) was applied to convert in vitro metabolic activity to in vivo hepatic clearance for comparison and applications in future models.

## 2 MATERIALS AND METHODS

### 2.1 Chemicals

Naloxone hydrochloride dihydrate (NX, ≥98%), naltrexone hydrochloride (NTX, ≥99%), fentanyl citrate (FEN, ≥98%), remifentanil hydrochloride (RMF, ≥97%), testosterone (TES, ≥98%), dextromethorphan hydrobromide (DM, ≥98%), (S)‐(+)‐mephenytoin (SM, ≥98%), potassium phosphate monobasic (≥99%), potassium phosphate dibasic (≥98%), and phosphate buffered saline (PBS) were obtained from Sigma‐Aldrich. Nalmefene hydrochloride (NMF, ≥99%) and nalmefene‐D_3_ (NMF‐D_3_, >98%) were obtained from Tocris Bioscience. Carfentanil citrate (CRF, >95%) was obtained from the chemical synthesis laboratory at the US Army Combat Capabilities Development Command Chemical Biological Center (APG, MD). Naloxone‐D_5_ (NX‐D_5_, ≥99%), naltrexone‐D_3_ (NTX‐D_3_, ≥99%), fentanyl‐D_5_ (FEN‐D_5_, ≥99), carfentanil‐D_5_ (CRF‐D_5_, ≥99%), testosterone‐D_3_ (TES‐D_3_, ≥99%), and dextromethorphan‐D_3_ (DM‐D_5_, ≥99%) were obtained from Cerilliant. Remifentanil‐D_5_ (RMF‐D_5_, >97%) was obtained from ClearSynth (Lower Hutt). Mephenytoin‐D_5_ (SM‐D_5_, >98%) was obtained from Santa Cruz Biotechnology. Formic acid (≥99%, Optima™ LC/MS grade), methanol (≥99.9%, Optima™ LC/MS grade), and dimethyl sulfoxide (DMSO, ≥99.9%, HPLC grade) were obtained from Fisher Scientific. Williams E media, fetal bovine serum (FBS), and primary hepatocyte plating and maintenance supplements were obtained from ThermoFisher. Trypsin (0.25%) containing 0.02% EDTA was obtained from Quality Biological.

### 2.2 Metabolism: recombinant CYPs

Individual CYPs with substantial predicted metabolic activity (>20% of total metabolism) were chosen for each test article based on predictions generated using ADMET Predictor (v. 10.0.0.11; Simulations Plus, Inc.).^35^ Total predicted metabolic activity and percent contributions for individual CYPs are given in Table S1. To summarize, all test articles were predicted to be metabolized via CYP3A4, while a select few were projected to undergo metabolism via multiple CYP isozymes (2C19 for NX and 2D6 for FEN and CRF).

Yeast expressing recombinant human CYP3A4, CYP2D6, and CYP2C19 (CypExpress™) were obtained from Sigma‐Aldrich and reconstituted in potassium phosphate buffer (100 mM, pH 7.4) to a final concentration of 50 mg/ml, according to the manufacturer's recommendations. Due to the poorly water‐soluble nature of test articles, concentrated solutions were prepared in DMSO to yield a 50× stock or 2% final DMSO concentration in the reaction mixture. Reactions were performed in a 24‐well plate to ensure sufficient agitation for optimal aeration of the reaction mixture. At the start of each reaction, 196 μl of reconstituted CypExpress™ and 4 μl of 50*x* test article were added to each well and incubated at 600 rpm and 30°C. To correlate CypExpress™ and CYP concentrations, standard substrates for each CYP (TES for CYP3A4, DM for CYP2D6, and SM for CYP2C19) were subjected to analogous reaction conditions for CYP quantification.^36^ All reactions were performed in duplicate. At the end of each predetermined time point (0, 0.5, 1, 2, and 4 h), reactions were quenched with the addition of 200 μl of ice‐cold methanol, and samples were centrifuged at 14 000 *g* for 10 min at room temperature to remove cellular debris. Supernatants were then stored at −20°C until LC–MS/MS analysis.

### 2.3 Metabolism: hepatic spheroids

Spheroid‐qualified human hepatocytes were obtained from ThermoFisher and cultured according to the manufacturer's recommendations. Briefly, cells were seeded at a density of 1500 viable cells per well onto Nunclon™ Sphera™‐treated U‐bottom 96‐well plates (ThermoFisher) pre‐wetted with plating media (Williams E media supplemented with 5% FBS, 15 mM HEPES, 2 mM, GlutaMAX™, 1 μM dexamethasone, 4 μg/ml human recombinant insulin, and 10 000 U/ml penicillin/streptomycin). Cells were then incubated at 37°C and 5% CO_2_ for 5 days undisturbed to enable spheroid aggregation, after which cells were maintained in serum‐free maintenance media (Williams E media supplemented with 15 mM HEPES, 2 mM GlutaMAX™, 0.1 μM dexamethasone,1.25 mg/ml bovine serum albumin, 6.25 μg/ml human recombinant insulin, 6.25 μg/ml human transferrin, 6.25, 5.35 μg/ml linoleic acid, 6.25 ng/ml selenous acid, and 10 000 U/ml penicillin/streptomycin).

Concentrated solutions of test articles were prepared in DMSO and subsequently diluted 50× in maintenance media. Cells were then dosed by removing half of the media in each well and replacing it with an equal volume of diluted test article (1% final DMSO concentration). Metabolic determinations in hepatic spheroids were performed in duplicate for each concentration and each time point. At the end of each predetermined time point (0, 1, 2, 4, and 24 h), reactions were quenched with the addition of 200 μl of ice‐cold methanol and centrifuged at 2500 *g* for 5 min at room temperature. Supernatants were collected and stored at −20°C until LC–MS/MS analysis.

To account for differences in spheroid aggregates, cell concentrations were determined after each time point to normalize substrate decay. Briefly, spheroids were washed with 150 μl of PBS and dissociated with the addition of 150 μl of trypsin for 15 min at 37°C and 200 rpm. Following trypsinization, manual agitation (i.e., vigorous pipetting for 1 min) was applied to fully dissociate spheroids, confirmed by light microscopy. Once a single cell suspension was attained, trypsin action was terminated with the addition of an equal volume of saline solution containing 10% FBS. Cell count was then determined using flow cytometry (Attune™ NxT acoustic focusing cytometer; ThermoFisher) with a 50 μl total acquisition volume per sample. Cell debris was excluded using a forward‐ and side‐scattered light gate (Figures S1–S3), from which cell count was calculated using the gated cell density and total cell suspension volume.

### 2.4 Sample preparation

Prior to sample extraction, samples were diluted 5‐ to 200‐fold in water to produce final analyte concentrations in the range of 5–500 ng/ml and prevent the loss of analytes during solid‐phase extraction (final methanol content <10%). Isotopically labeled standards (NX‐D_5_, NTX‐D_3_, NMF‐D_3_, FEN‐D_5_, RMF‐D_5_, CRF‐D_5_, TES‐D_3_, DM‐D_3_, and SM‐D_5_) were spiked into each sample to produce a final concentration of 50–100 ng/ml. Prepared samples were then extracted by solid‐phase extraction (SPE) using a positive pressure manifold (ASPEC Positive Pressure Manifold; Gilson Inc.). Oasis HLB 96‐well plates containing 30 mg sorbent per well (Waters Corporation) were first conditioned with 1 ml of methanol, followed by 1 ml of water, after which samples were loaded onto the plate and subsequently washed with 0.5 ml of water twice. Analytes were then eluted with 1 ml methanol divided over two additions. Eluates were collected in 96‐well collection plates (Phenomenex), and eluents were evaporated under a dry nitrogen stream (Biotage SPE Dry 96 Solvent Evaporator; Biotage). After thorough drying, samples were reconstituted in 1 ml of water containing 0.1% formic acid for LC–MS/MS analysis.

### 2.5 LC–MS/MS

Analyte concentrations were determined in triplicate. Chromatographic separation was performed on an Agilent 1290 Infinity liquid chromatograph (Agilent Technologies) using a Halo C18 column (2.7 μm, 2.1 × 50 mm) (Advanced Materials Technology). Analytes were separated using gradient programs optimized for each target (see Tables S2–S4) with a flow rate of 500 μl/min, an injection volume of 5 μl, and mobile phases composed of (A) water with 0.2% formic acid and (B) methanol with 0.2% formic acid. Observed retention times (*t*_*R*_) are given in Table 1 for all targets and internal standards.

**TABLE 1:** MRM transitions and optimized MS parameters for test articles and internal standards

Compound	*t*_R_ (min)	Q1 (Da)	Q3 (Da)	CE (V)	CXP (V)
NX	0.92	328.0	253.2^a^	35.0	12
			212.0^b^	35.0	14
NX‐D5	0.90	333.0	258.0^a^	38.0	30
			212.0^b^	52.0	27
NTX	1.07	342.0	270.0^a^	36.0	9
			282.0^b^	40.0	17
NTX‐D3	1.05	345.0	270.0^a^	40.0	31
			285.0^b^	39.0	34
			322.0^a^	28.0	40
NMF	1.56	340.0	268.0^b^	38.0	31
			252.0^b^	55.0	29
NMF‐D3	1.54	343.0	325.0^a^	28.0	36
			268.0^b^	39.0	31
FEN	2.07	337.0	188.0^a^	31.0	23
			105.0^b^	41.0	13
FEN‐D5	2.02	343.0	189.0^a^	32.5	24
			105.0^b^	42.0	15
			317.0^a^	21.0	39
RMF	1.91	377.0	228.0^b^	26.0	28
			113.0^b^	33.0	13
			322.0^a^	23.0	35
RMF‐D5	1.89	382.0	228.0^b^	27.5	27
			113.0^b^	36.0	18
			202.2^a^	35.0	14
CRF	2.53	395.2	246.2^b^	29.0	11
			279.3^b^	32.0	19
			335.0^b^	24.0	23
			207.0^a^	34.0	23
CRF‐D5	2.49	400.0	284.0^b^	32.0	38
			246.0^b^	29.0	27
			340.0^b^	26.0	45
TES	4.03	289.0	109.0^a^	29.0	13
			97.0^b^	30.0	15
TES‐D3	4.02	292.0	109.0^a^	28.0	13
			97.0^b^	27.0	15
DM	2.50	272.0	215.0^a^	33.0	25
			147.0^b^	40.0	18
DM‐D5	2.49	275.0	215.0^a^	34.0	26
			147.0^b^	41.0	19
SM	2.81	219.0	117.0^a^	33.0	12
			134.0^b^	21.0	16
SM‐D5	2.79	224.0	122.0^a^	28.0	15
			139.0^b^	23.0	18

Abbreviations: CE, collision energy; CRF, carfentanil; CXP, collision exit potential; DM, dextromethorphan; FEN, fentanyl; NMF, nalmefene; NTX, naltrexone; NX, naloxone; RMF, remifentanil; SM, S‐mephenytoin; TES, testosterone; *t*_R_, retention time.

^a^ Quantifier.

^b^ Qualifier.

Analytes were detected with tandem mass spectrometry using a Sciex 6500 QTRAP triple quadrupole mass spectrometer (Sciex) operated in positive electrospray ionization mode with multiple reaction monitoring (MRM) and the following ion source parameters: curtain gas, 35 psi; ion spray voltage, 5500 V; ion source temperature 550°C; nebulizer gas; 70 psi; and heater gas, 60 psi. For each target analyte, multiple transitions were monitored, with one transition selected for quantification, and the remaining transitions serving as qualifiers to confirm analyte identity (Table 1). Analyte‐specific parameters, specifically collision energy (CE) and collision exit potential (CXP), were optimized for maximum signal and are given in Table 1. Declustering potential, entrance potential, and collision‐assisted dissociation gas did not exhibit analyte‐specific effects and were set at 50, 10 V, and low, respectively, for all analytes.

### 2.6 Data analysis

Cell counts were analyzed using Attune™ NxT Software (v. 3.1.2; ThermoFisher) to determine cell density and exclude cell debris. Analyte peak areas for quantifiers and qualifiers were integrated using Analyst Instrument Control and Data Processing Software (v. 1.6.2; Sciex). Initial reaction velocities and Michaelis–Menten parameters were determined using GraphPad Prism (GraphPad Prism v 8.4.3; GraphPad Software Inc.). Initial velocities were evaluated using an exponential plateau fit on substrate consumed versus time data and subsequently plotted against substrate concentration to determine Michaelis‐ Menten parameters.

Intrinsic clearances (CL_int_) were calculated for a 70 kg human (see Table S5 for liver properties) using experimentally determined Michaelis–Menten parameters and the correlation between hepatic CL_int_ and enzyme kinetics as derived in Choi et al.^37^

(1) $${\mathrm{CL}}_{\mathrm{int}}=\frac{V_{\max}}{K_{m}}A$$

where *V*_max_ is the maximal rate of reaction, *K*_m_ is the Michaelis constant, and *A* is the amount of CYPs or hepatocytes in the liver tissue.

The liver's contribution to systemic clearance, also termed hepatic clearance (CL_H_), was calculated using a well‐stirred clearance model:

(2) $${\mathrm{CL}}_{H}=\frac{Q_{H}f_{u,p}{\mathrm{CL}}_{\mathrm{int}}}{Q_{H}+f_{u,p}{\mathrm{CL}}_{\mathrm{int}}}$$

where *Q*_H_ is the hepatic blood flow, *f*_u,p_ is the fraction unbound (Table S6), and CL_int_ is the intrinsic clearance. In this model, the liver is approximated as a well‐mixed compartment with a fixed drug concentration.^37^

## 3 RESULTS

### 3.1 Metabolism via recombinant CYPs

**FIGURE 1:**
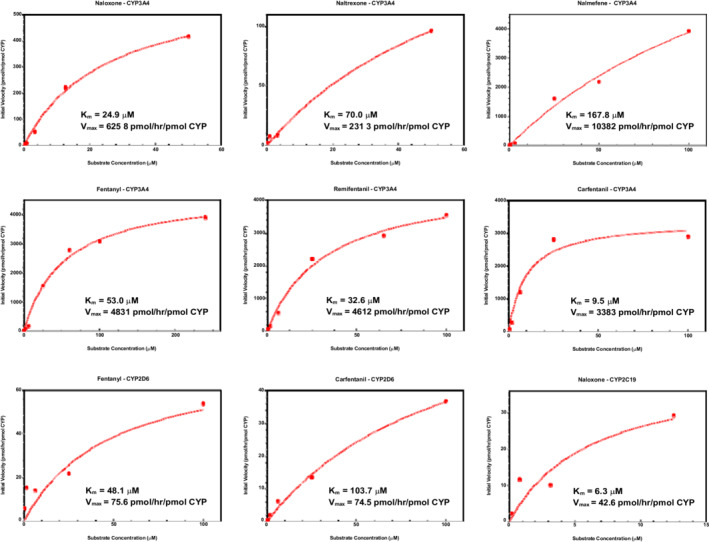
Michaelis–Menten kinetics for the metabolism of opioids and opioid antagonists by specific cytochrome P450 enzymes (CYPs). Yeast expressing recombinant human CYP3A4, CYP2D6, and CYP2C19 were reconstituted in potassium phosphate buffer (100 mM, pH 7.4) to a final concentration of 50 mg/ml. Reactions were initiated with the addition of substrate, incubated at 600 rpm and 30°C, and terminated after 0, 0.5, 1, 2, and 4 h. Substrate consumption was determined using LC–MS/MS. Initial velocities were determined using an exponential plateau fit and are expressed in units of substrate consumed per hour per pmol of CYP, with CYP concentrations quantified using standard substrates for each CYP (TES for CYP3A4, DM for CYP2D6, and SM for CYP2C19). *n* = 2 for each time point at each concentration

Metabolism of opioids and opioid antagonists via individual CYPs with considerable predicted metabolic activity (>20% of total metabolism) is shown in Figure 1. Rates of substrate consumption were evaluated for multiple substrate concentrations and followed typical Michaelis‐Menten kinetics, for all combinations of CYPs and substrate investigated. *V*_max_ and *K*_m_ were derived using the Michaelis–Menten equation and are given in Table S7 for each substrate‐CYP pair. CL_int_ were calculated from in vitro parameters using an in vitro‐in vivo correlation and are shown in Figure 3 in red (see Table S8 for numerical values and individual CYP contributions). CYP3A4 metabolism was characterized for all substrates and found to be dominant compared to all other CYPs investigated. In general, opioids and opioid antagonists exhibited different levels of metabolic activity via CYP3A4, with higher CL_int_ observed for opioids compared to antagonists (avg_opioid_ = 1545 L/h vs. avg_antagonist_ = 237 L/h). CYP2C19 metabolism was also characterized for NX and found to have minimal contributions compared to CYP3A4 (CYP2C19 = ~3% of CYP3A4). Similarly, FEN and CRF were subjected to CYP2D6 metabolism and found to have largely negligible metabolic activity via that pathway (CYP2D6 = ≤1% of CYP3A4).

### 3.2 Metabolism via hepatic spheroids

**FIGURE 2:**
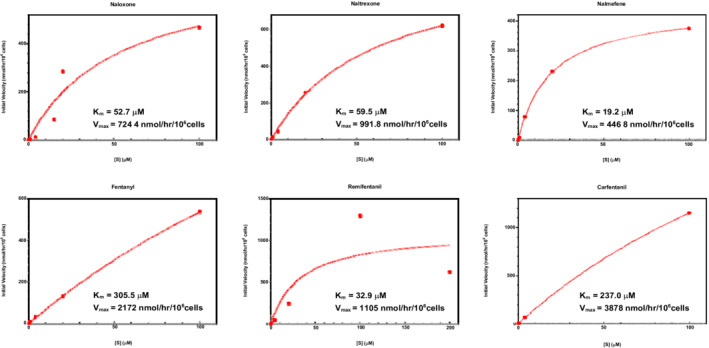
Michaelis–Menten kinetics for the metabolism of opioids and opioid antagonists in spheroid‐qualified human hepatocytes. Cells were seeded at a density of 1500 viable cells per well and exposed on the seventh day, following 5 days of undisturbed spheroid aggregation and an additional 2 days in serum‐free maintenance media. Reactions were initiated with the addition of substrate, incubated at 37°C and 5% CO_2_, and terminated after 0, 1, 2, 4, and 24 h. Initial velocities were determined using an exponential plateau fit and are expressed in units of substrate consumed per hour per 10^6^ cells. Substrate consumption was determined using LC‐MS/MS. Cell concentrations were determined using flow cytometry following spheroid dissociation at the end of each time point. *n* = 2 for each time point at each concentration

**FIGURE 3:**
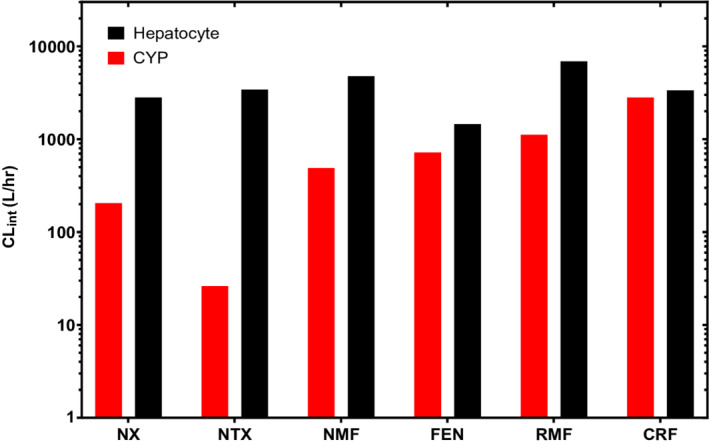
Intrinsic clearance (CL_int_) of opioids (fentanyl, FEN; remifentanil, RMF; carfentanil, CRF) and opioid antagonists (naloxone, NX; naltrexone, NTX; nalmefene, NMF) in yeast expressing specific cytochrome P450 enzymes (CYPs, red) and human hepatocytes (black). Intrinsic clearance was calculated for an average human (liver tissue weight of 1800 g) using experimentally determined Michaelis–Menten parameters (*V*_max_ and *K*_m_) as derived previously.^37^ See Table S7 for numerical values and contributions from individual CYPs

Metabolism of opioids and opioid antagonists was characterized in hepatic spheroids and is shown in Figure 2, with fitted *V*_max_ and *K*_m_ values given in Table S9. Although all substrates exhibited typical Michaelis–Menten kinetics, data spread was considerably greater when compared with those observed in CYP metabolism. CL_int_ were derived analogously using Michaelis–Menten parameters and an in vitro‐in vivo correlation and are shown in Figure 3 in black (see Table S8 for numerical values). In terms of hepatic metabolism, all substrates exhibited fairly comparable metabolic activities, with all CL_int_ spanning the same order of magnitude and comparable average CL_int_ for both categories of substrate (avg_opioid_ = 3888 L/h vs. avg_antagonist_ = 3657 L/h). Nevertheless, antagonist metabolism was characterized by a narrower range of CL_int_ (span of 1948 L/h), whereas opioid metabolism exhibited a broader CL_int_ range (span of 5416 L/h); the overall slowest and fastest metabolism was observed within the opioids investigated, for FEN and RMF, respectively. CL_H_ was also calculated using experimentally obtained hepatic CL_int_ and literature reported *f*_u,p_ and are given in Table 2. In general, antagonists were characterized by higher CL_H_ (avg_antagonist_ = 1.20 L/h/kg) compared to that of opioids (avg_opioid_ = 1.04 L/h/kg), even though this distinction was not apparent for CL_int_.

**TABLE 2:** The liver's contribution to systemic clearance (CL_H_) for opioids and opioid antagonists as determined using experimental Michaelis‐Menton parameters for human hepatocytes and a well‐stirred clearance model

Compound	CLH (L/h/kg)
NX	1.09
NTX	1.12
NMF	1.12
FEN	0.55
RMF	0.81
CRF	0.63

Abbreviations: CRF, carfentanil; FEN, fentanyl; NMF, nalmefene; NTX, naltrexone; NX, naloxone; RMF, remifentanil.

## 4 DISCUSSION

Opioid abuse is an ongoing public health concern, exacerbated by the emergence of more potent synthetic opioids, specifically FEN‐class opioids.^1^, ^2^, ^4^, ^5^ Existing competitive antagonists (e.g., NX, NTX, NMF) have been proven effective against morphine and FEN‐induced respiratory depression; however, efficacy against more potent opioids is not well‐established.^10^, ^12^, ^13^, ^14^, ^15^, ^18^ In particular, renarcotization remains a relevant issue due to the relatively short half‐lives of available opioid antagonists.^19^ To characterize the stability of antagonists in the context of opioids, metabolic studies have been conducted; however, these studies often employed in vitro systems comprising different ensembles of metabolic pathways and relevant transport phenomena, complicating direct comparisons.^27^, ^28^, ^29^ A comprehensive metabolic study utilizing the same in vitro systems across multiple opioids and opioid antagonists would yield valuable information on relative metabolic stability and thereby facilitate the development of antagonists that are efficacious against potent synthetic opioids. To this end, metabolic clearance was characterized in human hepatocytes and yeast expressing individual recombinant CYPs for FEN‐class opioids and common opioid antagonists.

For both in vitro systems investigated, metabolism followed typical Michaelis–Menten kinetics for all substrates (Figures 1 and 2). Unsurprisingly, a larger data spread was observed in hepatocytes, likely due to the more complex and heterogeneous nature of whole cell metabolism compared to a single enzymatic reaction (i.e., individual CYPs). For instance, metabolism in the cell is governed by intracellular drug concentrations, which dictate the amount of drug available to be metabolized. Slight variations in this property may arise due to a number of factors that influence passive and active transport of substrates across the cell membrane.^38^ As an added factor, hepatocytes were also cultured to form spheroid aggregates in an effort to mimic cell–cell interactions present in the in vivo environment, and even though metabolic rates were normalized to cell counts, the spheroid morphology presents an additional source of variability.^39^ In addition to data spread, notable disparities in the CL_int_ calculated from Michaelis–Menten parameters (Figure 3) may also be attributed to fundamental differences between the two in vitro systems. As expected, intrinsic metabolic activity as measured in hepatic spheroids was higher than the aggregate of CYP contributions for all opioids and antagonists investigated, due to the inclusion of additional metabolic pathways. These distinctions emphasize the need to understand distinguishing features of in vitro models, as one may consider multiple in vitro systems for a complete metabolic representation or select a single in vitro system in an effort to maintain consistency and comparability.

Although only a single enzymatic reaction is represented, CYPs play a crucial role in drug and xenobiotic metabolism, with five isozymes (1A2, 2C9, 2C19, 2D6, and 3A4) responsible for the oxidation and metabolism of more than half of all marketed drugs.^24^, ^40^ Metabolic simulations for opioids and opioid antagonists identified CYP3A4, CYP2D6, and CYP2C19 as major contributors to overall metabolism (Table S1). Of these, only the 3A4 isozyme was predicted and experimentally confirmed to significantly process all substrates of interest (Table S8). This is anticipated for opioids, which are known to be predominantly metabolized via CYP‐mediated oxidation.^25^ On the other hand, even though opioid antagonists undergo glucuronidation more readily, substantial CYP3A4 activity was observed, albeit to a lesser degree than that measured for opioids. This additional metabolic activity from phase I CYP‐mediated oxidation may culminate in the short half‐lives of opioid antagonists relative to FEN‐class opioids, which are not known to undergo glucuronidation to any significant degree.^25^ Such consequences may present an important consideration for novel antagonist design, as the apparent ubiquity of CYP metabolism may hinder antagonist action if peripheral metabolic pathways are simultaneously present.

Select opioids and opioid antagonists were projected to undergo metabolism via multiple CYP isozymes, in this case, 2C19 for NX and 2D6 for FEN and CRF, in conjunction with 3A4 (Table S1). For these substrates, measured CYP3A4 metabolic rates were considerably dominant in terms of intrinsic enzymatic activity, with CL_int,3A4_ at least an order of magnitude greater than that of other isozymes. Moreover, relative contributions of individual CYPs were markedly different from simulations, with far greater metabolic activity via CYP3A4 than predicted (Table S8). In fact, oxidation mediated by either CYP2D6 or CYP2C19 was practically negligible (<5% and <1% of CYP3A4 activity, respectively, Table S8), even though both were expected to have metabolic activity equal to, if not greater than, CYP3A4 (Table S1). These inconsistencies are not too surprising given the reported performance of both models used to determine CYP contributions: site of metabolism model (67.5%–79.2% correct predictions) and enzyme kinetics model (CYP3A4, *R* ^2^ = .544–.667; CYP2D6, *R* ^2^ = .546–.820; and CYP2C19, *R* ^2^ = .711–.816).^35^ These results further highlight the need to determine metabolic contributions experimentally in order to confirm or better inform computational models. Moreover, in a large study on marketed drugs, the 3A4 isozyme was found to be responsible for nearly half of all CYP‐mediated oxidation.^24^ The evident prevalence of CYP3A4 metabolism, coupled with the heightened CL_int_ contrary to predictions, presents a possible premise on which to conceptualize new opioid antagonists. Given the predominant metabolic pathways for opioids (i.e., phase I CYP‐mediated oxidation), molecular structures capable of binding at opioid receptors, but solely metabolized via other isozymes or phase II pathways, may produce efficacious countermeasures due to enhanced longevity. While confirmatory studies are necessary, these screening parameters could be readily adapted in computational drug discovery for countermeasure development.

Contrary to the distinct separation between opioids and antagonists observed for CYP metabolism, intrinsic metabolic activities as measured in hepatic spheroids were largely comparable for all substrates (Figure 3). Only minute differences were discernable in terms of the spans of CL_int_ calculated for each class, with antagonists characterized by a considerably narrower range. This consistency in measured metabolic activity may be due to fundamental similarities in chemical structure. While agonists investigated share a common backbone, the overall degree of molecular similarity, as often quantified by the Tanimoto coefficient (*T*_C_), is far greater amongst the morphine‐based antagonists (*T*_c_ >0.9 for antagonists vs. *T*_c_ = 0.6–0.9 for agonists, Tables S10 and S11).^41^ Given the nature of enzyme‐substrate specificity, it may not be uncommon for structurally analogous molecules to share metabolic pathways, undergo similar biotransformations, and ultimately exhibit comparable metabolic rates.^42^ The largely similar CL_int_ observed for highly structurally analogous antagonists supports this theory and may provide the underlying basis for a structure–activity relationship for opioid/opioid antagonist metabolism. These relationships would be invaluable for future antagonist development, especially in the early stages of drug discovery.

Finally, CL_H_ were calculated using experimentally determined CL_int_, as hepatic clearance is ultimately governed by drug bioavailability. Notably, CL_H_ for opioids were lower than those for antagonists despite having similar, if not slightly higher, CL_int_ (avg_opioid_ = 1.04 L/h/kg; avg_antagonist_ = 1.20 L/h/kg). This discrepancy underscores the importance of considering additional ADMET (absorption, distribution, metabolism, excretion, and toxicity) properties in congruence with intrinsic metabolism, as CL_H_ is a function of blood flow, fraction of unbound drug in the plasma (*f*_u,p_), and CL_int_ As such, to attain physiologically relevant results, factors affecting drug distribution must be considered, and in this case, lower *f*_u,p_ for opioids (avg = 0.20) resulted in diminished hepatic metabolism even though they were characterized by higher CL_int_. This outcome presents an additional element on which novel antagonists could be formulated, as *f*_u,p_ is closely related to lipophilicity, with more lipophilic molecules exhibiting enhanced protein binding and therefore lower *f*_u,p_.^43^ Given this relationship, piecewise structural modifications could be implemented to improve the lipophilicity of potential opioid countermeasures in an effort to extend antagonist effect. In theory, these physicochemical considerations, along with the desired metabolic features discussed previously (e.g., low CYP3A4 activity), should produce antagonists that are effective even against more potent opioids and should be further investigated to confirm the validity of utilizing these elements as a foundation for future drug development studies.

The results presented herein represent a comprehensive metabolic study utilizing the same in vitro system across multiple opioids and opioid antagonists, with hopes to facilitate the development of antagonists that are efficacious against potent synthetic opioids. While metabolism via the CYP system and hepatic spheroids were explored, metabolism via other major metabolizing pathways (e.g. glucuronidation) or other in vitro systems (e.g. human liver microsomes) were not investigated in this study. Future studies incorporating the impact of glucuronidation and other metabolic pathways, such as metabolism via enzymes in plasma, and in either well established models, such as liver microsomes, or advanced in vitro systems (e.g. “liver‐on‐a‐chip” platforms) may produce more robust data sets in‐line with complete metabolic representation. Future investigations will center on of improving the screening criteria established here to better aid the development of countermeasures against synthetic opioids.

## AUTHOR CONTRIBUTIONS

*Participated in research design*: Tuet, Pierce, diTargiani, McCranor, Wong. *Conducted experiments*: Tuet, Pierce, Conroy, Tressler. *Contributed new reagents or analytic tools*: Vignola, diTargiani. *Performed data analysis*: Tuet, Pierce. *Wrote or contributed to the writing of the manuscript*: Tuet, McCranor.

## FUNDING INFORMATION

This work was supported by an interagency agreement (AOD2106‐001‐00000) between the NIH Office of the Director (OD) and the U.S. Army Medical Research Institute of Chemical Defense under the oversight of the Chemical Countermeasures Research Program (CCRP) within the Office of Biodefense Research (OBRS) at the National Institute of Allergy and Infectious Diseases (NIAID/NIH). W.Y.T., S.A.P., and M.C. were supported in part by an appointment to the Department of Defense (DOD) Research Participation Program administered by the Oak Ridge Institute for Science and Education (ORISE) through an interagency agreement between the U.S. Department of Energy (DOE) and the DOD. ORISE is managed by Oak Ridge Associated Universities (ORAU) under DOE contract number DE‐SC0014664.

## DISCLOSURE

The authors declare no competing interest.

## ETHICS STATEMENT

The views expressed herein are those of the authors and do not reflect the official policy of the Department of Army, Department of Defense, or the U.S. Government.

## Supporting information


Appendix S1

## Data Availability

The data that support the findings of this study are available from the corresponding author upon reasonable request.
