# Supplementary material for: Metabolic clearance of select opioids and opioid antagonists using hepatic spheroids and recombinant cytochrome P450 enzymes
Source: Pharmacol Res Perspect. 2022 Aug 31;10(5):e01000. doi: 10.1002/prp2.1000 (PMC9433823; doi:10.1002/prp2.1000)
Supplement: Supplementary file 1 — Appendix S1 [file PRP2-10-e01000-s001.docx]

## **Supplemental Information for**

## **Metabolic clearance of select opioids and opioid antagonists using hepatic spheroids and recombinant cytochrome P450 enzymes**

Wing Y. Tuet, Samuel A. Pierce, Matthieu Conroy, Justin N. Vignola, Justin Tressler, Robert C. diTargiani, Bryan J. McCranor, and Benjamin Wong

**Table S1.** Total predicted metabolic activity and percent contribution of individual CYPs.

| Compound | **Total metabolic activity**  **(µL/min/mg HLM)** | **CYP3A4**  **(%)** | **CYP2D6**  **(%)** | **CYP2C19**  **(%)** |
| --- | --- | --- | --- | --- |
| NX | 25.9 | 40.2 | - | 58.3 |
| NTX | 8.9 | 87.6 | - | - |
| NMF | 11.2 | 71.4 | 10.7 | - |
| FEN | 45.9 | 34.6 | 48.6 | 13.3 |
| RMF | 115.2 | 95.9 | - | 3.8 |
| CRF | 75.2 | 58.8 | 41.1 | - |

**Table S2.** Gradient program for the elution of NX, NTX, RMF, TES, and SM.

| t  (min) | **Mobile phase composition** | |
| --- | --- | --- |
|  | **A (%)** | **B (%)** |
| 0 | 90 | 10 |
| 5 | 5 | 95 |
| 5.5 | 5 | 95 |
| 6 | 90 | 10 |
| 7.5 | 90 | 10 |

**Table S3.** Gradient program for the elution of NMF.

| t  (min) | **Mobile phase composition** | |
| --- | --- | --- |
|  | **A (%)** | **B (%)** |
| 0 | 80 | 20 |
| 0.5 | 80 | 20 |
| 2.5 | 75 | 25 |
| 3 | 10 | 90 |
| 3.5 | 10 | 90 |
| 4 | 80 | 20 |
| 5.5 | 80 | 20 |

**Table S4.** Gradient program for the elution of FEN, CRF, and DM.

| t  (min) | **Mobile phase composition** | |
| --- | --- | --- |
|  | **A (%)** | **B (%)** |
| 0 | 70 | 30 |
| 0.5 | 70 | 30 |
| 3.5 | 60 | 40 |
| 4 | 10 | 90 |
| 4.5 | 10 | 90 |
| 5 | 70 | 30 |
| 6 | 70 | 30 |

**Table S5.** Liver properties for a 70 kg human.

| Property | **Value** | **Reference** |
| --- | --- | --- |
| Tissue weight  (g) | 1800 | Davies and Morris (1993) |
| Blood flow  (mL/min/kg) | 20.7 | Davies and Morris (1993) |
| Microsomal protein content  (mg MP/g liver tissue) | 39.46 | Zhang et al., (2015) |
| CYP3A4 expression  (pmol CYP/mg MP) | 111 | Inoue et al., (2006) |
| CYP2D6 expression  (pmol CYP/mg MP) | 8 | Inoue et al., (2006) |
| CYP2C19 expression  (pmol CYP/mg MP) | 14 | Inoue et al., (2006) |
| Hepatocellularity  (cells/g liver tissue) | 113.5 x 10^6^ | Arias (1988); Wilson et al., (2003) |

**Table S6.** Fraction unbound in human plasma for opioids and opioid antagonists.

| Compound | ***f_u,p_*** | **Reference** |
| --- | --- | --- |
| NX | 0.54 | Lombardo et al., (2018) |
| NTX | 0.79 | Lombardo et al., (2018) |
| NMF | 0.65 | Lombardo et al., (2018) |
| FEN | 0.15 | Kalvass et al., (2007); Feasel et al., (2018); Lombardo et al., (2018) |
| RMF | 0.30 | Lombardo et al., (2018) |
| CRF | 0.14 | Feasel et al., (2018) |

**Table S7.** Michaelis-Menten kinetics for the metabolism of opioids and opioid antagonists via recombinant CYPs.

| CYP | **Compound** | **V_max_**  **(pmol/hr/pmol CYP)** | **K_m_**  **(µM)** |
| --- | --- | --- | --- |
| 3A4 | NX | 625.8 ± 56.3 | 24.9 ± 5.1 |
|  | NTX | 231.3 ± 127.0 | 70.0 ± 65.3 |
|  | NMF | 10382 ± 3170 | 167.8 ± 74.5 |
|  | FEN | 4831 ± 356 | 53.0 ± 10.9 |
|  | RMF | 4612 ± 398 | 32.6 ± 7.7 |
|  | CRF | 3383 ± 345 | 9.5 ± 3.5 |
| 2D6 | FEN | 75.6 ± 29.5 | 48.1 ± 42.9 |
|  | CRF | 74.5 ± 13.5 | 103.7 ± 33.2 |
| 2C19 | NX | 42.6 ± 22.3 | 6.3 ± 7.5 |

**Table S8.** Intrinsic clearance of opioids and opioid antagonists.

| Compound | **CL_int_ (L/hr)** | | | |
| --- | --- | --- | --- | --- |
|  | **CYP3A4^a^** | **CYP2D6^b^** | **CYP2C19^c^** | **Hepatocyte^d^** |
| NX | 197.8 | - | 6.76 | 2809 |
| NTX | 26.1 | - | - | 3406 |
| NMF | 487.8 | - | - | 4757 |
| FEN | 718.5 | 0.894 | - | 1453 |
| RMF | 1115 | - | - | 6868 |
| CRF | 2802 | 0.408 | - | 3343 |

**Table S9.** Michaelis-Menten kinetics for the metabolism of opioids and opioid antagonists in hepatic spheroids.

| Compound | **V_max_**  **(nmol/hr/10^6^ cells)** | **K_m_**  **(µM)** |
| --- | --- | --- |
| NX | 724.4 ± 222.0 | 52.7 ± 34.1 |
| NTX | 991.8 ± 48.1 | 59.5 ± 6.4 |
| NMF | 446.8 ± 12.1 | 19.2 ± 1.6 |
| FEN | 2172 ± 183 | 305.5 ± 33.7 |
| RMF | 1105 ± 457 | 32.9 ± 48.6 |
| CRF | 3878 ± 1192 | 237.0 ± 103.5 |

**Table S10.** Tanimoto coefficient for all agonist pairs.

| Tanimoto coefficient (T_C_) | | | |
| --- | --- | --- | --- |
|  | **CRF** | **RMF** | **FEN** |
| CRF | 1 | 0.908 | 0.738 |
| RMF | - | 1 | 0.667 |
| FEN | - | - | 1 |

**Table S11.** Tanimoto coefficient for all antagonist pairs.

| Tanimoto coefficient (T_C_) | | | |
| --- | --- | --- | --- |
|  | **NX** | **NTX** | **NMF** |
| NX | 1 | 0.948 | 0.904 |
| NTX | - | 1 | 0.956 |
| NMF | - | - | 1 |

**Table S12.** Intrinsic clearance as compared to previous studies.

| Intrinsic clearance (CL_int_) | | | | |
| --- | --- | --- | --- | --- |
|  | **This study** | | **Literature** | |
|  | **CYPs** | **Hepatic spheroids** |  |  |
| NX | 205^a^ | 2809^b^ | 306^b^ | Sohlenius-Sternbeck et al., (2010) |
|  |  |  | 60^c^ | Sohlenius-Sternbeck et al., (2010) |
|  |  |  | 956 – 992^b^ | Blanchard et al., (2005) |
|  |  |  | 71 – 3591^d^ | Blanchard et al., (2005) |
|  |  |  | 184 – 1324^e^ | Blanchard et al., (2005) |
| NTX | 26^a^ | 3406^b^ | 21 – 158^c^ | Porter et al., (2000) |
| NMF | 488^a^ | 4757^b^ | N/A | |
| FEN | 719^a^ | 1453^b^ | N/A | |
| RMF | 1115^a^ | 6868^b^ | N/A | |
| CRF | 2803^a^ | 3343^b^ | 381^c^ | Feasel et al., (2016) |

^a^recombinant CYPs; ^b^hepatocytes (cryopreserved); ^c^human liver microsomes; ^d^hepatocytes (freshly suspended); ^e^hepatocytes (conventional primary cultures)


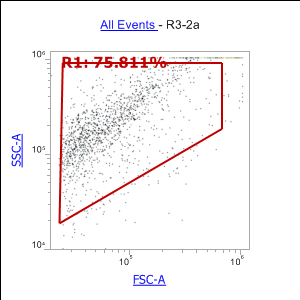


**Figure S1.** Hepatocyte gate used to quantify cells in dissociated spheroids. Forward- and side-scatter were used to exclude cell debris. On average, 75 ± 4% of total cells were included in the defined gate for each sample.

**Figure S2.** Exponential plateau fits on substrate consumed vs. time data for the metabolism of select opioids and opioid antagonists via recombinant CYPs.

**Figure S3.** Exponential plateau fits on substrate consumed vs. time data for the metabolism of select opioids and opioid antagonists via hepatic spheroids.

**References**

Arias, I. (1988). Introduction. The Liver: Biology and Pathobiology. I. Arias, H. Popper, W. Jokoby, D. Schacter and D. Shafritz. New York, Raven Press**:** 3-10.

Blanchard, N., E. Alexandre, C. Abadie, T. Lave, B. Heyd, G. Mantion, D. Jaeck, L. Richert and P. Coassolo (2005). "Comparison of clearance predictions using primary cultures and suspensions of human hepatocytes." Xenobiotica **35**(1): 1-15.

Davies, B. and T. Morris (1993). "Physiological parameters in laboratory animals and humans." Pharm Res **10**(7): 1093-1095.

Feasel, M. G., R. J. Lawrence, R. L. Kristovich, A. Wohlfarth and M. A. Huestis (2018). Translational human health assessment of carfentanil using an experimentally refined PBPK model, ARMY EDGEWOOD CHEMICAL BIOLOGICAL CENTER APG MDNational Inst on Drug Abuse.

Feasel, M. G., A. Wohlfarth, J. M. Nilles, S. Pang, R. L. Kristovich and M. A. Huestis (2016). "Metabolism of Carfentanil, an Ultra-Potent Opioid, in Human Liver Microsomes and Human Hepatocytes by High-Resolution Mass Spectrometry." The AAPS Journal **18**(6): 1489-1499.

Inoue, S., E. Howgate, K. Rowland-Yeo, T. Shimada, H. Yamazaki, G. Tucker and A. Rostami-Hodjegan (2006). "Prediction of in vivo drug clearance from in vitro data. II: potential inter-ethnic differences." Xenobiotica **36**(6): 499-513.

Kalvass, J. C., T. S. Maurer and G. M. Pollack (2007). "Use of Plasma and Brain Unbound Fractions to Assess the Extent of Brain Distribution of 34 Drugs: Comparison of Unbound Concentration Ratios to in Vivo P-Glycoprotein Efflux Ratios." Drug Metabolism and Disposition **35**(4): 660-666.

Lombardo, F., G. Berellini and R. S. Obach (2018). "Trend Analysis of a Database of Intravenous Pharmacokinetic Parameters in Humans for 1352 Drug Compounds." Drug Metabolism and Disposition **46**(11): 1466-1477.

Porter, S. J., A. A. Somogyi and J. M. White (2000). "Kinetics and inhibition of the formation of 6β‐naltrexol from naltrexone in human liver cytosol." British journal of clinical pharmacology **50**(5): 465-471.

Sohlenius-Sternbeck, A. K., L. Afzelius, P. Prusis, J. Neelissen, J. Hoogstraate, J. Johansson, E. Floby, A. Bengtsson, O. Gissberg, J. Sternbeck and C. Petersson (2010). "Evaluation of the human prediction of clearance from hepatocyte and microsome intrinsic clearance for 52 drug compounds." Xenobiotica **40**(9): 637-649.

Wilson, Z. E., A. Rostami-Hodjegan, J. L. Burn, A. Tooley, J. Boyle, S. W. Ellis and G. T. Tucker (2003). "Inter-individual variability in levels of human microsomal protein and hepatocellularity per gram of liver." Br J Clin Pharmacol **56**(4): 433-440.

Zhang, H., N. Gao, X. Tian, T. Liu, Y. Fang, J. Zhou, Q. Wen, B. Xu, B. Qi, J. Gao, H. Li, L. Jia and H. Qiao (2015). "Content and activity of human liver microsomal protein and prediction of individual hepatic clearance in vivo." Scientific Reports **5**(1): 17671.
